# Evaluation of a Metaverse-Based Refracture Prevention Education Program for Nurses Applying the ARCS Model: A Quasi-Experimental Study

**DOI:** 10.3390/healthcare14203408

**Published:** 2026-10-13

**Authors:** Bona Park, Myung Sook Park

**Affiliations:** 1 Infection Control Department, Seran General Hospital, Seoul 03182, Republic of Korea; pbn1208@kku.ac.kr; 2 Department of Nursing, Konkuk University, Chungju 27478, Republic of Korea

**Keywords:** refracture, older adults, nurses, learning motivation, clinical judgment, metaverse, ARCS model, nursing education

## Abstract

**Highlights:**

**What are the main findings?**

- In individual-level analyses, the ARCS-based metaverse program was associated with higher post-intervention motivation and greater changes in self-efficacy, self-reported nursing performance, and self-reported clinical judgment. The nursing performance finding was sensitive to the analytical model used.
- Knowledge showed no significant group-by-time interaction; findings were sensitive to analytic assumptions and the four-ward design.

**What are the implications of the main findings?**

- A browser-based 2D metaverse platform may offer a flexible educational option for shift-working nurses.
- The findings may inform the development of continuing education related to fracture liaison service coordinator competencies.

**Abstract:**

**Background/Objectives**: In aging societies, refractures after a first fragility fracture are a major public health burden, and nurses play a central role in their prevention. This study evaluated an ARCS-based metaverse refracture prevention education program for nurses. **Methods**: A quasi-experimental pre-test–post-test study included 63 nurses from 4 non-randomly allocated wards at a single general hospital (experimental group [EG], *n* = 32; control group [CG], *n* = 31). The EG completed eight ZEP-based sessions over four weeks; the CG received the same topics through video lectures. Post-intervention learning motivation was compared using the Mann–Whitney U test. Self-efficacy, self-reported nursing performance, self-reported clinical judgment, and knowledge were analyzed using group-by-time interactions. **Results**: Motivation was higher in the EG (U = 138, *p* < 0.001). Individual-level interactions were significant for self-efficacy (F = 7.87, *p* = 0.007), performance (F = 8.00, *p* = 0.006), and clinical judgment (F = 10.96, *p* = 0.002) but not knowledge (F = 3.31, *p* = 0.074). Four of six clinical-judgment subdimensions met the Bonferroni criterion. Performance and knowledge findings differed under baseline adjustment, and pre–post findings were not significant in exploratory ward-level comparisons. **Conclusions**: The program was associated with favorable short-term educational outcomes. The non-randomized four-ward design and sensitivity to analytic assumptions limit causal and practical conclusions.

## 1. Introduction

The global population is aging rapidly [1]. Osteoporosis increases bone fragility and the likelihood of fragility fractures, which can result from low-energy trauma such as a fall from standing height [2]. After an initial fragility fracture, the risk of a subsequent fracture is particularly elevated during the following one to two years [3,4].

To prevent subsequent fractures, the Fracture Liaison Service (FLS)—a coordinated model encompassing risk assessment, pharmacologic treatment, rehabilitation, and nutritional management—has been widely adopted, and a recent meta-analysis reported a lower risk of secondary fragility fractures among FLS participants [5]. In many countries, nurses serve as FLS coordinators and perform functions including patient identification, risk assessment, education, and discharge planning [6]. In Korea, limited awareness of FLS and uncertainty regarding the coordinator role have been identified as implementation barriers [7,8]. Nursing care is integral to the management of patients with fragility fractures [9], but few educational interventions have focused specifically on refracture prevention for nurses. Because clinical nurses often work rotating three-shift schedules, they face constraints on the time and place available for conventional in-person education. Consequently, web-based, self-directed learning has been proposed as a practical approach for continuing nursing education [10]. Building on this foundation, immersive and interactive digital learning approaches have been explored to further enhance learner engagement [11].

Metaverse-based education has emerged as one approach to providing interactive digital learning. Avatar-based environments can support self-directed participation and interaction among learners [12,13]. Because such environments depend on active learner participation, motivational design may be relevant. Keller’s ARCS model addresses learning motivation through four components—Attention, Relevance, Confidence, and Satisfaction—and has been applied in nursing education [14,15]. A browser-based 2D metaverse platform has also been used to deliver core nursing-skill education [16].

Applications of the metaverse in nursing education have expanded, but reviews indicate that many studies have focused on basic nursing skills or individual simulation scenarios [13]. Head-mounted virtual reality may be constrained by hardware requirements, cost, and cybersickness [17]. In contrast, browser-based 2D platforms may offer a practical alternative for repeated access, although direct comparative evidence remains limited [16]. ARCS-based teaching in nursing education has been associated with improved learning motivation and related outcomes [15]. However, an ARCS-based metaverse program focused on refracture prevention for nurses has not yet been evaluated. The program assessed in this study was developed in a companion study [18].

Therefore, this study aimed to evaluate an ARCS-based metaverse refracture prevention education program for nurses in relation to learning motivation, learning self-efficacy, self-reported nursing performance, self-reported clinical judgment, and refracture prevention knowledge. It was hypothesized that the experimental group would have higher post-intervention motivation and subscale scores and greater pre–post changes in the other outcomes and clinical-judgment subdimensions. No single primary outcome was prospectively designated.

## 2. Materials and Methods

### 2.1. Study Design

This study used a quasi-experimental, non-equivalent control group pre-test–post-test design to examine changes in outcomes associated with a metaverse-based refracture prevention education program for nurses. Trial registration was not applicable to this quasi-experimental educational study.

### 2.2. Participants

**Figure 1:**
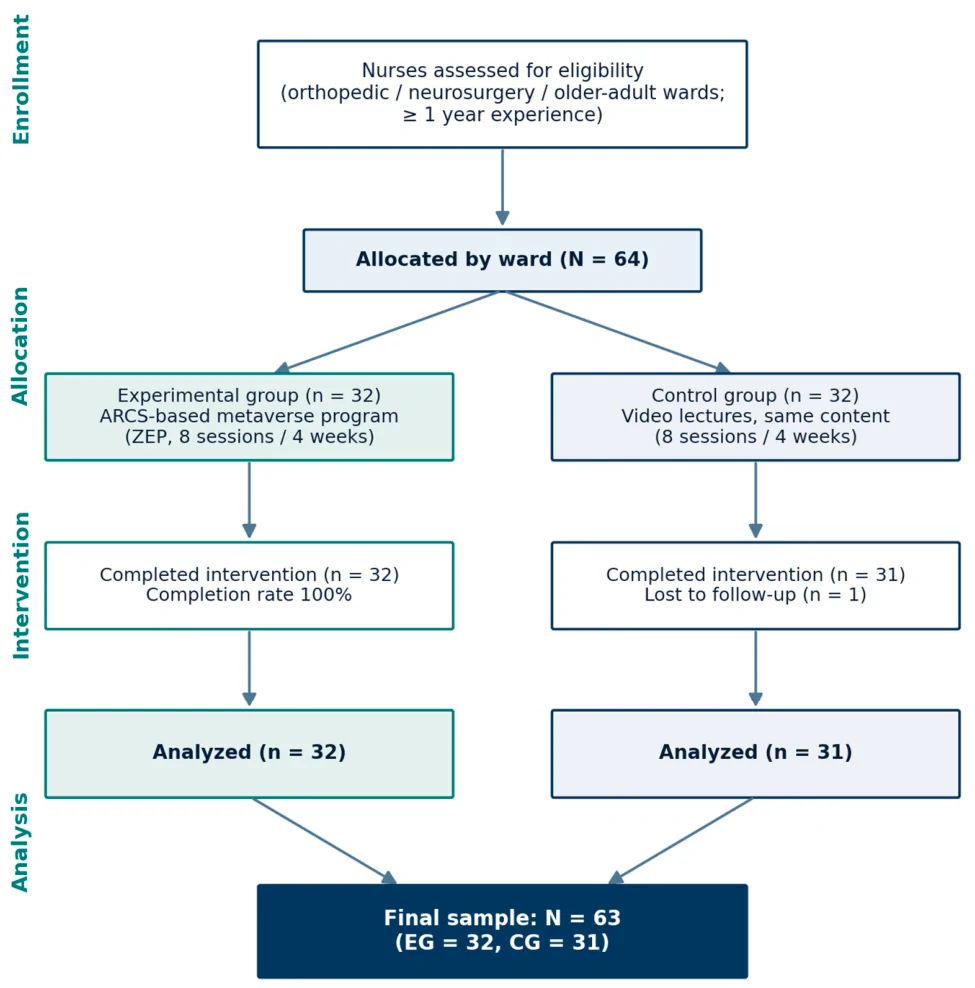
Flow diagram of participant enrollment, allocation, intervention, and analysis.

Participants were nurses at a general hospital in Seoul, Republic of Korea, who had at least one year of experience in orthopedic surgery, neurosurgery, or wards caring for older adults; were able to use internet-based media; and consented to participate. The sample-size calculation assumed a two-sided independent-samples *t* test (α = 0.05, power = 0.90, standardized mean difference d = 0.85), requiring 31 nurses per group. The effect-size assumption followed Lee [19], who drew on the ARCS meta-analysis by Kang and Shon [20]; it was not specific to a refracture prevention outcome. No intra-cluster correlation or design effect was included. Allowing for attrition, 32 nurses were allocated per group; 1 CG participant was lost to follow-up, leaving 63 for analysis (Figure 1). Allocation was non-randomized at the ward level to minimize contact between conditions. Each department contributed one intervention ward and one control ward: neurosurgery, EG 19 and CG 16; orthopedic surgery, EG 13 and CG 15.

### 2.3. The Education Program

**Figure 2:**
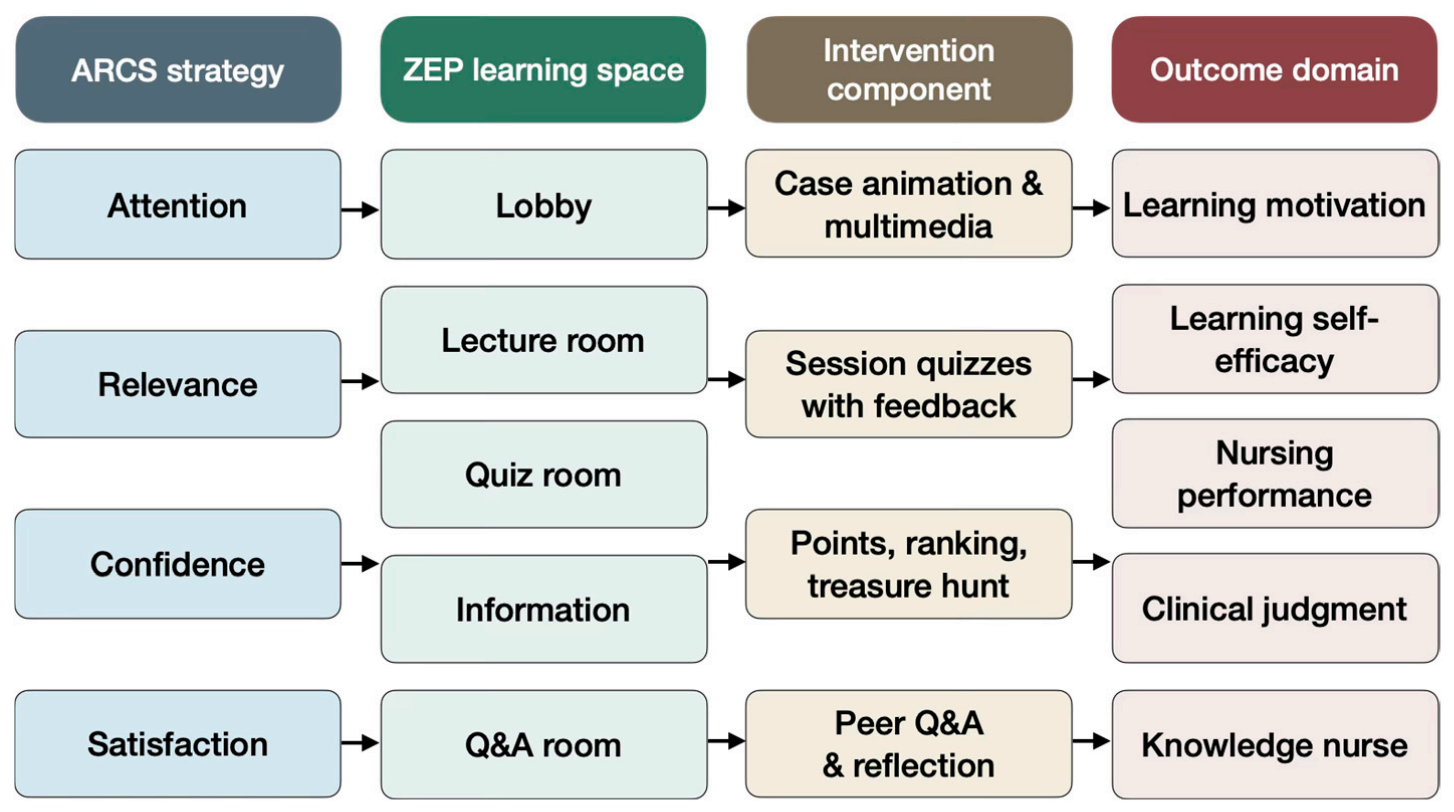
Structure of the ARCS-based metaverse refracture prevention education program, linking ARCS strategies, ZEP learning spaces, intervention components, and outcome domains. Nursing performance and clinical judgment refer to self-reported outcomes; knowledge refers to refracture prevention knowledge.

The intervention was an eight-session program (twice weekly, 25 min per session, over four weeks) developed using Keller’s ARCS model and implemented on the 2D metaverse platform ZEP; the development procedures and content validity are reported separately [18]. Briefly, the program comprised five interactive spaces (lobby, lecture room, quiz room, information room, and Q&A room) and addressed osteoporosis, bone density testing, fracture and refracture treatment, prevention exercises, medications, nutritional management, fall prevention, and communication strategies (Figure 2). Each ARCS component was operationalized through multimedia animations and clinical case scenarios (Attention and Relevance), session quizzes with feedback (Confidence), and gamified rewards, including point collection and rank-based prizes (Satisfaction). The EG participated through avatar-based interaction in the metaverse, whereas the CG received the same eight topics through video lectures. Throughout the intervention period, the researcher monitored session-by-session access and completion in both groups and provided individual reminders to participants who had not completed a session. The two nurses involved in preliminary usability testing did not participate in this evaluation. All analyzed participants completed eight sessions (EG 32/32; CG 31/31), corresponding to 100% and 96.9% of those initially allocated. Both groups received monitoring and reminders, with video completion defined by recorded viewing progress of at least 95%. Comparable total time spent on all learning activities was not available.

### 2.4. Measurements

Learning motivation was assessed after the intervention using the 36-item Instructional Materials Motivation Survey (IMMS) [21], with Attention (12 items), Relevance (9), Confidence (9), and Satisfaction (6). Korean nursing applications have described the Jang adaptation [22]. The administered questionnaire used five-point responses and reverse-scoring of negatively worded items (total range 36–180; α = 0.97); its construct validity was not independently established in this sample. Learning self-efficacy was measured with a 10-item scale (range 10–50; α = 0.96) [23]. Self-reported refracture prevention nursing performance was measured with 10 researcher-developed items covering risk assessment, prevention, communication, and discharge education, reviewed by four nursing professors. Items were scored 0–3 (total 0–30); relevance-based I-CVI values were 0.75–1.00 and S-CVI/Ave was 0.95 (α = 0.89). Self-reported clinical judgment was measured with a 23-item, 6-dimension scale (range 23–115; α = 0.98) [24]. Refracture prevention knowledge was assessed with 26 items (correct = 1, incorrect = 0; total 0–26; scale-level content validity index = 0.93). Higher scores indicate higher levels of each variable.

### 2.5. Ethical Considerations and Data Collection

This study was conducted in accordance with the Declaration of Helsinki and approved by the Institutional Review Board of Konkuk University (protocol code 7001355-202404-HR-775). Participants were informed of the purpose, anonymity, and the right to withdraw, and they provided written informed consent. Pre-test data were collected before the intervention, and post-test data were collected immediately after the four-week intervention via self-report questionnaires distributed through online survey links. Analyses included the 63 participants with available pre–post data; no missing item imputation was used in the final dataset.

### 2.6. Data Analysis

Data were analyzed using IBM SPSS Statistics 26.0. Baseline characteristics were summarized descriptively and compared using independent-samples *t* tests for continuous variables and Fisher’s exact tests for categorical variables; nonsignificant baseline tests were not interpreted as equivalence. Shapiro–Wilk tests examined pre-test, post-test, and change-score distributions. Despite some non-normality, a common 2 × 2 repeated-measures ANOVA was used for the four pre–post outcomes, considering the balanced group sizes and two measurement occasions. Group-by-time interactions estimated between-group differences in change; these analyses did not adjust standard errors forward dependence. Post-only motivation was compared using the Mann–Whitney U test. Effect estimates and 95% confidence intervals (CIs), partial eta-squared for interactions, and rank-biserial correlations for motivation were reported. Bonferroni correction was applied separately to four motivation subscales and six clinical judgment subdimensions. The five main domains were not collectively adjusted and are interpreted as exploratory. Baseline-adjusted ANCOVA was applied to all four pre–post outcomes as a sensitivity analysis, irrespective of baseline significance. Additional calculations included ward-level comparisons and descriptive intracluster correlations. Supplementary Tables S1–S5 provide distributions, effect estimates, multiplicity results, and sensitivity methods. Tests were two-sided (α = 0.05).

## 3. Results

### 3.1. Baseline Characteristics

The mean age was 37.7 years in the EG and 36.3 years in the CG. Baseline characteristics are presented in Table 1. Baseline self-reported nursing performance was lower in the EG (20.31 ± 6.87) than in the CG (23.84 ± 5.91); this imbalance and the non-randomized allocation were considered in interpretation.

### 3.2. Program Outcomes

Post-intervention learning motivation was higher in the EG than in the CG (167.69 ± 11.59 vs. 143.23 ± 19.73; U = 138, Z = −4.93, *p* < 0.001). The rank-biserial correlation was 0.722 (95% CI, 0.520 to 0.891). All four motivation subscales met the Bonferroni criterion (Table 2; Supplementary Table S1).

Significant group-by-time interactions were observed for learning self-efficacy (F(1,61) = 7.87, *p* = 0.007, partial η^2^ = 0.114), self-reported refracture prevention nursing performance (F(1,61) = 8.00, *p* = 0.006, partial η^2^ = 0.116), and self-reported clinical judgment (F(1,61) = 10.96, *p* = 0.002, partial η^2^ = 0.152). The corresponding between-group differences in pre-to-post change were 5.66 (95% CI, 1.63 to 9.70), 5.14 (95% CI, 1.51 to 8.78), and 16.64 (95% CI, 6.59 to 26.69), respectively. For refracture prevention knowledge, the group-by-time interaction was not statistically significant (F(1,61) = 3.31, *p* = 0.074, partial η^2^ = 0.051; difference in change, 1.27; 95% CI, −0.13 to 2.67) (Table 3). Baseline-adjusted ANCOVA yielded *p* = 0.089 for performance and *p* = 0.003 for knowledge; self-efficacy and clinical judgment remained significant (Supplementary Table S4). Exploratory ward-level comparisons were not significant for the four pre–post outcomes (*p* = 0.095–0.218; Supplementary Table S5).

After Bonferroni correction, group-by-time interactions remained significant for integrated data analysis, evidence-based intervention, interprofessional collaboration, and patient-centered care, but not for evaluation and reflection on interventions or collaboration among peer nurses (Table 4; Supplementary Table S3).

## 4. Discussion

Post-intervention motivation was higher in the EG, including all four subscales after Bonferroni correction. Interactive multimedia may have supported attention, clinically relevant scenarios may have supported relevance [14], and feedback and rewards may have contributed to confidence and satisfaction [15]. These mechanisms were not tested. The intervention combined ARCS design, metaverse delivery, clinical cases, gamification, feedback, and peer interaction; the contribution of any single component cannot be isolated.

A browser-based 2D platform was selected to reduce the hardware and setup demands associated with head-mounted virtual reality [13,17]. All analyzed participants in both groups completed eight sessions. This finding should be interpreted cautiously because the researcher monitored participation and reminded participants in either group who had not completed a session; therefore, completion may reflect both program characteristics and administrative support. Gamified elements, including point collection, ranking rewards, and a researcher-developed treasure hunt activity, were incorporated to support participation. Gamification has shown beneficial effects in nursing education, although the present study did not isolate the contribution of individual game elements [25]. The avatar-mediated Q&A room also provided opportunities for peer interaction and experience sharing, consistent with qualitative reports of supportive online peer-learning experiences in nursing education [26]. The program content addressed FLS coordinator functions such as patient identification, risk assessment, education, and discharge planning [6,8].

In individual-level analyses, learning self-efficacy improved more in the experimental group than in the control group. Previous metaverse and immersive simulation studies have reported mixed findings for self-efficacy [27,28], whereas a metaverse-based handoff program reported higher handoff self-efficacy but no between-group advantage in handoff competence [29]. Differences in intervention dose, content, participants, and study design may partly account for these variations. The eight-session format, clinically linked content, feedback, and rewards may have contributed to the present result [15,25], but these mechanisms were not tested directly.

Self-reported nursing performance showed a greater mean change in the EG in the individual-level analysis, but this difference was not significant after baseline adjustment. This result is therefore sensitive to the analytical model and should not be interpreted as established clinical behavior change. Case-based learning may support perceived application of content. Digital literacy and metaverse perspectives have been associated with academic motivation [30]. In metaverse-based operating-room patient safety education, improvements were observed within both groups without a significant between-group advantage [31].

Self-reported clinical judgment showed a greater individual-level mean change in the EG; four of six subdimensions met the Bonferroni criterion. Case-based quizzes may have supported review of clinical cues and application of content. Virtual simulation studies have examined critical thinking and clinical decision making outcomes [27,32], but differences in constructs and tools limit direct comparison. The 16.64-point difference in change on the 23–115 scale describes magnitude, not an established clinically important benefit.

Knowledge increased in both groups, but its group-by-time interaction was not significant; the significant baseline-adjusted result indicates model sensitivity. Several limitations should be considered. Single-site recruitment limits generalizability. Non-random allocation across only four wards permits selection and ward effects; individual-level CIs and *p*-values may overstate precision, and exploratory ward-level analyses did not support the pre–post findings at α = 0.05. With two wards per condition, these sensitivity analyses cannot establish robust cluster-adjusted inference. Nursing performance and clinical judgment were self-reported. High internal consistency does not establish construct validity and may reflect item redundancy; ceiling effects may also restrict discrimination. No single primary outcome was prospectively designated, and multiplicity across the main domains remains a limitation. Outcomes were measured immediately after the intervention; long-term retention, objective performance, and patient outcomes were not assessed. Statistical significance should not be equated with educational importance.

## 5. Conclusions

The ARCS-based metaverse refracture prevention education program was associated with higher post-intervention motivation and favorable individual-level changes in self-efficacy, self-reported nursing performance, and self-reported clinical judgment. The nursing performance finding, however, was sensitive to the analytical model used. Knowledge did not show a significant group-by-time interaction. The non-randomized four-ward design and sensitivity to analytic assumptions make these findings preliminary. Studies with more wards, longer follow-up, objective performance measures, and patient outcomes are needed.

## Figures and Tables

**Table 1:** Baseline characteristics of participants (N = 63).

Characteristics (Categories)	EG (*n* = 32)	CG (*n* = 31)	*t*	*p*
Gender (Male/Female)	2/30	3/28	—	0.672
Age (years), M ± SD	37.72 ± 8.71	36.26 ± 8.67	0.67	0.507
Education (≥Bachelor’s), *n* (%)	25 (78.1)	26 (83.8)	—	0.750
Department (neurosurgery), *n* (%)	19 (59.4)	16 (51.6)	—	0.616
Total clinical experience (years)	12.75 ± 8.63	11.35 ± 9.60	0.61	0.546
Position (Staff nurse), *n* (%)	27 (84.4)	28 (90.3)	—	0.708
Prior RP education (Yes), *n* (%)	8 (25.0)	8 (25.8)	—	1.000

EG, experimental group; CG, control group; RP, refracture prevention; M, mean; SD, standard deviation. Continuous variable *p*-values use independent-samples *t* tests; categorical variable *p*-values use two-sided Fisher’s exact tests.

**Table 2:** Between-group differences in learning motivation after the intervention.

Variable	EG (M ± SD)	CG (M ± SD)	Z	*p*
Learning motivation (total)	167.69 ± 11.59	143.23 ± 19.73	−4.93	<0.001
Attention	57.19 ± 3.89	47.94 ± 8.23	−4.70	<0.001
Relevance	42.19 ± 2.78	36.45 ± 5.22	−4.59	<0.001
Confidence	39.88 ± 3.69	34.71 ± 4.54	−4.29	<0.001
Satisfaction	28.44 ± 2.40	24.13 ± 4.07	−4.54	<0.001

EG, experimental group (*n* = 32); CG, control group (*n* = 31); M, mean; SD, standard deviation. Z: Mann–Whitney U test; displayed *p*-values are unadjusted. The four subscales were assessed against α = 0.0125. U statistics and median [Q1, Q3] values are in Supplementary Table S1.

**Table 3:** Pre–post outcomes in the experimental and control groups.

Variable	Group	Pre (M ± SD)	Post (M ± SD)	F(1,61)	*p*
Learning self-efficacy	EG	42.75 ± 7.01	49.22 ± 2.42	7.87	0.007
	CG	42.48 ± 5.47	43.29 ± 6.81		
Self-reported nursing performance	EG	20.31 ± 6.87	26.84 ± 4.44	8.00	0.006
	CG	23.84 ± 5.91	25.23 ± 4.13		
Self-reported clinical judgment	EG	91.69 ± 15.86	109.97 ± 7.60	10.96	0.002
	CG	94.74 ± 14.06	96.39 ± 14.95		
Refracture prevention knowledge	EG	19.06 ± 2.02	21.69 ± 2.10	3.31	0.074
	CG	18.39 ± 2.09	19.74 ± 2.52		

EG (*n* = 32), experimental group; CG (*n* = 31), control group. Values are unadjusted M ± SD. F(1,61): individual-level group-by-time interaction, without ward adjustment. Nursing performance and clinical judgment were self-reported. Effect estimates and distribution summaries appear in Supplementary Table S2.

**Table 4:** Between-group differences in clinical-judgment subdimensions (pre-to-post change).

Subdimension	EG Δ (M ± SD)	CG Δ (M ± SD)	F(1,61)	*p*
Integrated data analysis	4.56 ± 4.15	0.48 ± 6.26	9.35	0.003
Evaluation and reflection on interventions	2.34 ± 2.47	0.84 ± 3.09	4.58	0.036
Evidence-based intervention	3.25 ± 3.08	0.00 ± 4.49	11.27	0.001
Interprofessional collaboration	2.72 ± 3.22	−0.42 ± 4.01	11.79	0.001
Patient-centered care	3.41 ± 3.81	0.65 ± 4.02	7.83	0.007
Collaboration among peer nurses	2.00 ± 2.57	0.10 ± 3.27	6.63	0.012

EG (*n* = 32), experimental group; CG (*n* = 31), control group; Δ, post-minus-pre change; F(1,61), individual-level group-by-time interaction without ward adjustment. Displayed *p*-values are unadjusted; the six subdimensions were assessed against α = 0.05/6. Bonferroni-adjusted *p*-values appear in Supplementary Table S3.

## Data Availability

De-identified data and analysis syntax may be requested from the corresponding author. Sharing is subject to a review of participant privacy, re-identification risk, and applicable institutional requirements; individual-level data are not publicly available.

## Supplementary Materials

The following supporting information can be downloaded at https://www.mdpi.com/article/10.3390/healthcare14203408/s1, Table S1: Learning motivation; Table S2: Pre–post outcomes; Table S3: Clinical judgment subdimensions; Table S4: Baseline adjusted sensitivity analysis; Table S5: Ward level sensitivity analysis.

## Abbreviations

The following abbreviations are used in this manuscript:

ARCS	Attention, Relevance, Confidence, Satisfaction
FLS	Fracture Liaison Service
EG	Experimental group
CG	Control group
ANCOVA	Analysis of covariance
