# Supplementary material for: Evaluation of a Metaverse-Based Refracture Prevention Education Program for Nurses Applying the ARCS Model: A Quasi-Experimental Study
Source: Healthcare (Basel). 2026 Oct 13;14(20):3408. doi: 10.3390/healthcare14203408 (PMC13704361; doi:10.3390/healthcare14203408)
Supplement: Supplementary file 1 [file healthcare-14-03408-s001.zip › healthcare-4520470-supplementary.pdf]

Supplementary statistical results

Manuscript healthcare-4520470

All individual-level analyses include 32 experimental-group and 31 control-group nurses. Positive contrasts favor the experimental group. Individual-level confidence intervals do not account for ward dependence. The analyses are exploratory.

Table S1 Learning motivation

Scale	EG median [Q1, Q3]	CG median [Q1, Q3]	U	Z	Raw p	Bonferroni p
Total	172.00 [160.25, 178.00]	145.00 [131.00, 154.00]	138.0	-4.926	<0.001	—
Attention	59.00 [55.25, 60.00]	47.00 [42.00, 52.00]	159.5	-4.696	<0.001	<0.001
Relevance	43.00 [40.25, 45.00]	36.00 [33.00, 40.00]	163.5	-4.593	<0.001	<0.001
Confidence	40.00 [37.00, 43.00]	35.00 [31.00, 37.00]	185.0	-4.292	<0.001	<0.001
Satisfaction	30.00 [27.25, 30.00]	24.00 [21.00, 27.00]	176.0	-4.535	<0.001	<0.001

Scale	Rank-biserial correlation [95% CI]	Mean difference [95% CI]
Total	0.722 [0.520, 0.891]	24.46 [16.23 to 32.69]
Attention	0.678 [0.464, 0.863]	9.25 [5.96 to 12.54]
Relevance	0.670 [0.455, 0.849]	5.74 [3.60 to 7.87]
Confidence	0.627 [0.401, 0.827]	5.17 [3.08 to 7.26]
Satisfaction	0.645 [0.432, 0.831]	4.31 [2.61 to 6.01]

Mann–Whitney tests use tie-adjusted asymptotic two-sided p-values without continuity correction. Rank-biserial correlation is 2P(EG > CG) + P(EG = CG) − 1. Its CI is a percentile bootstrap interval from 10,000 independent within-group resamples (seed 20261001), not a ward bootstrap. Mean-difference CIs use the Welch standard error and degrees of freedom; these summarize a different estimand from the rank test. Quartiles use the (n + 1)p interpolation convention. Bonferroni adjustment applies only to the four subscales (α = 0.0125).

Table S2 Pre–post outcomes

Outcome	Group (n)	Pre median [Q1, Q3]	Post median [Q1, Q3]
Learning self-efficacy	EG (32)	42.50 [39.25, 49.00]	50.00 [50.00, 50.00]
Learning self-efficacy	CG (31)	41.00 [39.00, 49.00]	43.00 [39.00, 50.00]
Self-reported nursing performance	EG (32)	20.50 [15.00, 26.75]	28.50 [25.00, 30.00]
Self-reported nursing performance	CG (31)	26.00 [19.00, 29.00]	27.00 [22.00, 29.00]
Self-reported clinical judgment	EG (32)	92.00 [81.75, 101.00]	114.50 [106.25, 115.00]
Self-reported clinical judgment	CG (31)	92.00 [88.00, 111.00]	95.00 [85.00, 108.00]
Refracture prevention knowledge	EG (32)	19.00 [18.00, 20.00]	21.50 [21.00, 23.00]
Refracture prevention knowledge	CG (31)	19.00 [17.00, 20.00]	20.00 [18.00, 22.00]

Outcome	Difference in change [95% CI]	F(1,61)	p	Partial η²
Learning self-efficacy	5.66 [1.63 to 9.70]	7.874	0.007	0.114
Self-reported nursing performance	5.14 [1.51 to 8.78]	8.000	0.006	0.116
Self-reported clinical judgment	16.64 [6.59 to 26.69]	10.957	0.002	0.152
Refracture prevention knowledge	1.27 [-0.13 to 2.67]	3.307	0.074	0.051

Mean-change contrasts are EG minus CG. With two time points, the group-by-time F equals the squared equal-variance t statistic comparing individual change scores. CIs use the pooled change-score variance and t(61); partial $\eta^2 = F/(F + 61)$. These four main-domain p-values are unadjusted. Mean and SD values appear in manuscript Table 3.

Table S3 Clinical judgment subdimensions

Subdimension	Difference in change [95% CI]	F(1,61)	Raw p	Bonferroni p
Integrated data analysis	4.08 [1.41 to 6.75]	9.346	0.003	0.020
Evaluation and reflection	1.51 [0.10 to 2.91]	4.576	0.036	0.219
Evidence-based intervention	3.25 [1.31 to 5.19]	11.273	0.001	0.008
Interprofessional collaboration	3.14 [1.31 to 4.97]	11.794	0.001	0.006
Patient-centered care	2.76 [0.79 to 4.73]	7.833	0.007	0.041
Peer nurse collaboration	1.90 [0.43 to 3.38]	6.631	0.012	0.075

The six comparisons form one Bonferroni family ($\alpha = 0.05/6$). Adjusted p-values were calculated from unrounded values. Four subdimensions meet this threshold. Mean-change CIs are unadjusted individual-level 95% intervals and are not simultaneous familywise intervals.

Table S4 Baseline adjusted sensitivity analysis

Outcome	Adjusted posttest difference [95% CI]	F(1,60)	p	Partial η^2
Learning self-efficacy	5.92 [3.34 to 8.50]	21.092	<0.001	0.260
Self-reported nursing performance	1.94 [-0.30 to 4.18]	2.988	0.089	0.047
Self-reported clinical judgment	13.35 [7.35 to 19.36]	19.813	<0.001	0.248
Refracture prevention knowledge	1.79 [0.63 to 2.96]	9.425	0.003	0.136

Each ANCOVA uses posttest score as the dependent variable, group as a factor, and the corresponding pretest score as a continuous covariate, with a common slope. Models were fitted for all four outcomes, not selected by baseline p-values. ANCOVA contrasts and change-score contrasts estimate different quantities. In this non-randomized study neither removes unmeasured confounding; these ANCOVAs also do not adjust for ward dependence. Performance and knowledge are sensitive to the choice of model.

Table S5 Ward level sensitivity analysis

Outcome	EG NS<br>n = 19	EG OS<br>n = 13	CG NS<br>n = 16	CG OS<br>n = 15
Learning motivation	167.05	168.62	143.69	142.73
Learning self-efficacy	8.42	3.62	2.50	-1.00
Self-reported nursing performance	8.00	4.38	0.06	2.80
Self-reported clinical judgment	23.37	10.85	-2.25	5.80
Refracture prevention knowledge	2.95	2.15	1.38	1.33

Entries are ward means of individual change scores for pre–post outcomes and posttest scores for motivation. NS, neurosurgery; OS, orthopedic surgery. One ward per condition in each department means that department × group identifies the four wards. Differences favor the intervention ward in both departments for each outcome, but their magnitudes vary.

Outcome	ICC	Ward mean contrast [95% CI]	t(2)	p
Learning motivation	-0.066	24.62 [20.68 to 28.56]	26.896	0.001
Learning self-efficacy	0.073	5.27 [-7.52 to 18.06]	1.772	0.218
Self-reported nursing performance	0.035	4.76 [-4.99 to 14.52]	2.100	0.171
Self-reported clinical judgment	0.074	15.33 [-16.69 to 47.36]	2.060	0.176
Refracture prevention knowledge	-0.047	1.20 [-0.51 to 2.91]	3.011	0.095

Ward comparisons are exploratory equal-variance t tests of two intervention ward means versus two control ward means, weighting wards equally ($df = 2$), without department pairing or baseline adjustment. They require parametric assumptions that cannot be evaluated reliably with four wards and are not randomization tests. The motivation p-value must not be interpreted as proof of a causal or robust cluster-adjusted effect. The four pre–post comparisons are not significant at 0.05.

Descriptive ICCs are conditional on treatment group, using a nested ANOVA method-of-moments estimate: $ICC = (MSB - MSW)/[MSB + (n0 - 1)MSW]$. MSB compares wards within treatment groups (2 df); MSW compares nurses within wards (59 df). The effective size coefficient n0 is $[N - \Sigma g(\Sigma j\ nj^2/Ng)]/2$. Negative estimates are shown rather than interpreted as protective clustering. With four wards these estimates are unstable and are not used to claim adequate power or to provide definitive corrected p-values.

Individual-level GLM and Mann–Whitney analyses were conducted in IBM SPSS Statistics. Additional calculations used the item-level data and the stated formulas. Bootstrap intervals and ward-level estimates are exploratory and do not establish causal effects.
